# Investigation of the Sympathetic Regulation in Delayed Onset Muscle Soreness: Results of an RCT

**DOI:** 10.3389/fphys.2021.697335

**Published:** 2021-09-16

**Authors:** Johannes Fleckenstein, Elmo W. I. Neuberger, Philipp Bormuth, Fabio Comes, Angelika Schneider, Winfried Banzer, Lorenz Fischer, Perikles Simon

**Affiliations:** ^1^Department of Sports Medicine and Exercise Physiology, Institute of Sports Sciences, Goethe-Universität Frankfurt am Main, Frankfurt am Main, Germany; ^2^Department of Sports Medicine, Rehabilitation and Disease Prevention, Institute of Sports Sciences, Johannes Gutenberg University Mainz, Mainz, Germany; ^3^Department of Psychiatry, Psychosomatic Medicine and Psychotherapy, Goethe-Universität Frankfurt am Main, Frankfurt am Main, Germany; ^4^Department of Orthopedics, Orthopedic University Hospital Friedrichsheim gGmbH, Goethe-Universität Frankfurt am Main, Frankfurt am Main, Germany; ^5^Institute of Occupational, Social and Environmental Medicine, Goethe-Universität Frankfurt am Main, Frankfurt am Main, Germany; ^6^Professor em. Interventional Pain Management, Neural Therapy, General Internal Medicine, University of Bern, Bern, Switzerland

**Keywords:** vegetative nervous system, pain therapy, neuroinflammation, neurophysiology, sports medicine, sympathetic maintained pain, cell-free DNA – cfDNA

## Abstract

Sports-related pain and injury is directly linked to tissue inflammation, thus involving the autonomic nervous system (ANS). In the present experimental study, we disable the sympathetic part of the ANS by applying a stellate ganglion block (SGB) in an experimental model of delayed onset muscle soreness (DOMS) of the biceps muscle. We included 45 healthy participants (female 11, male 34, age 24.16 ± 6.67 years [range 18–53], BMI 23.22 ± 2.09 kg/m^2^) who were equally randomized to receive either (i) an SGB prior to exercise-induced DOMS (preventive), (ii) sham intervention in addition to DOMS (control/sham), or (iii) SGB after the induction of DOMS (rehabilitative). The aim of the study was to determine whether and to what extent sympathetically maintained pain (SMP) is involved in DOMS processing. Focusing on the muscular area with the greatest eccentric load (biceps distal fifth), a significant time × group interaction on the pressure pain threshold was observed between preventive SGB and sham (*p* = 0.034). There was a significant effect on pain at motion (*p* = 0.048), with *post hoc* statistical difference at 48 h (preventive SGB Δ1.09 ± 0.82 cm VAS vs. sham Δ2.05 ± 1.51 cm VAS; *p* = 0.04). DOMS mediated an increase in venous cfDNA -as a potential molecular/inflammatory marker of DOMS- within the first 24 h after eccentric exercise (time effect *p* = 0.018), with a peak at 20 and 60 min. After 60 min, cfDNA levels were significantly decreased comparing preventive SGB to sham (unpaired *t*-test *p* = 0.008). At both times, 20 and 60 min, cfDNA significantly correlated with observed changes in PPT. The 20-min increase was more sensitive, as it tended toward significance at 48 h (*r* = 0.44; *p* = 0.1) and predicted the early decrease of PPT following preventive stellate blocks at 24 h (*r* = 0.53; *p* = 0.04). Our study reveals the broad impact of the ANS on DOMS and exercise-induced pain. For the first time, we have obtained insights into the sympathetic regulation of pain and inflammation following exercise overload. As this study is of a translational pilot character, further research is encouraged to confirm and specify our observations.

## Introduction

Acute pain is generally accompanied by acute inflammation, a protective response involving immune cells, blood vessels, and molecular mediators, which is physiologically thought to eliminate the initial cause of cell injury and initiate tissue repair. Inflammatory mediators are able to bind to nociceptors in the peripheral nervous system innervating areas such as fascia and muscle (Ji et al., 2016). They induce vasodilation, extravasation of plasma proteins, and the release of further chemical mediators (Rittner and Stein, 2005). Collectively, they sensitize normally high threshold nociceptors and awaken silent nociceptors so that they respond to normally innocuous stimulation (Jorum et al., 2007; Chapman et al., 2008). Sympathetic nerve terminals contribute to this sensitization by releasing norepinephrine and prostanoids (Pezet and McMahon, 2006). This interaction is also referred to as the inflammatory reflex of the autonomic nervous system (ANS) (Tracey, 2002).

The autonomic nervous system (ANS) is a functional unit consisting of the sympathetic and the parasympathetic nervous system (SNS and PNS), which is divided into a central (brain and spinal cord) and a peripheral part. Being an information and control system, the ANS balances all organ systems against each other, maintaining homeostasis. For this reason, it is logical that the ANS is also involved in pain, inflammation, and immune processes in a “coordinating” way and plays an important role in microcirculation (Elenkov et al., 2000; Tracey, 2002, 2009; Jänig, 2006; Schaible et al., 2011; Janig, 2014).

Delayed onset muscle soreness (DOMS) is a painful state in the skeletal muscle that often occurs following unaccustomed or eccentric exercise (Macintyre et al., 1995), and has been widely used as an experimental model in sports medicine. In addition, DOMS has been used as a translational model, which gives clinical insights into the complex interactions underlying acute sports-related pain (Fleckenstein et al., 2017b). DOMS is most likely initiated by mechanical stretching of the muscle, elongating the weakest sarcomere first (Morgan, 1990), with repetitive stretches to pronounce injury (Morgan and Proske, 2004). Besides the mechanical trauma, i.e., lactic acid accumulation, muscle spasm, and connective tissue damage, inflammation and enzyme or electrolyte efflux contribute to the occurrence of DOMS (for review: Duncan and Jackson, 1987; Lewis et al., 2012; Mizumura and Taguchi, 2016). While DOMS refers to perceived pain and discomfort at an early stage (approx. 48 h), the term exercise-induced muscle damage (EIMD) has been elaborated to express an inflammation-induced exacerbation with dysfunction and prolonged healing (Friden and Lieber, 2001; Fleckenstein et al., 2017b).

Recently, it has been shown that cell-free DNA (cfDNA) is a pro-inflammatory load dependent marker of aerobic and strength exercise (Breitbach et al., 2012; Haller et al., 2017). For strength training, elevated baseline cfDNA values have even been described as a potential marker for overtraining (Fatouros et al., 2006) and have been reported to occur the first 2 days following strength exercise in unaccustomed participants, while acute responses accumulated over the course of strength exercise sets within 10–60 min (Tug et al., 2017). Moreover, cfDNA has been implicated as an early predictor of muscle performance decrement following muscle damage (Andreatta et al., 2018). The ubiquitous sympathetic part of the ANS is specifically involved in these processes. Since microtraumas and inflammation seem to play a role in athletic overload (Seidel et al., 2012; Wilke et al., 2017), we investigated the association of alterations in cfDNA as a secondary outcome upon studying the association of the SNS with DOMS. As the return of inflamed tissues to homeostasis has been heavily linked to the resolution of acute inflammation (Serhan et al., 2008), we believe that a therapeutically decreased sympathetic activity may result in less pain intensity and duration in acute conditions accompanied by reduced levels in cfDNA.

The stellate ganglion (SG) is located at about the level of the 1st rib in the lamina prevertebralis fasciae cervicalis. Its feeding neurons originate from the core areas of the intermediolateral nucleus from C8 to Th5. The efferents of the SG affect the equilateral upper body quadrant, involving partial functions in the brain, heart, lung, upper extremity, musculature, skin, blood vessels including microcirculation, endothelial function, immune system, and inflammatory system (e.g., Jänig, 2006; Janig, 2014; Ding et al., 2019). The regulation of the immune and inflammatory system as well as pain by means of an stellate ganglion block (SGB) is not only peripheral but also central by influencing the above mentioned autonomic centers in the brain and is therefore not only bound to the upper body quarter, although is probably strongest there (Puente de la Vega Costa et al., 2016; Fischer et al., 2021).

We hypothesize that prolonged DOMS seem to be related to SNS activity, since blocking SNS activity rather reduces both sensory thresholds and function. Interrupting the inflammatory reflex with the injection of the anti-inflammatory local anesthetic procaine has been described as being a clinical approach to restore the physiologic conditions of the tissue (Cassuto et al., 2006). By temporarily disabling the SNS using a SGB, we are trying to determine whether and to what extent sympathetically maintained pain (SMP), and the kinetics of cfDNA, are involved in an experimental model of delayed onset muscle soreness (DOMS) of the biceps muscle.

## Materials and Methods

This monocentric randomized controlled trial was conducted at the Department of Sports Medicine, Institute of Sports Sciences, Goethe-University Frankfurt am Main, Germany. The study has been approved by the Ethics Committee of the University of Frankfurt (ref. 490/15) and has been registered at the German Clinical Trials Register (ref. DRKS00010623). Exclusion criteria were the lack of legal capacity, history of hypersensitivity to local anesthetics, simultaneous participation in another clinical trial or participation in any clinical trial involving administration of an investigational medicinal product within 30 days prior to the beginning of the clinical trial, physical or psychiatric conditions interfering with this study (e.g., pain, neuromuscular disease, or sensibility disorders), and known or persistent abuse of medication, drugs, or alcohol.

**FIGURE 1:**
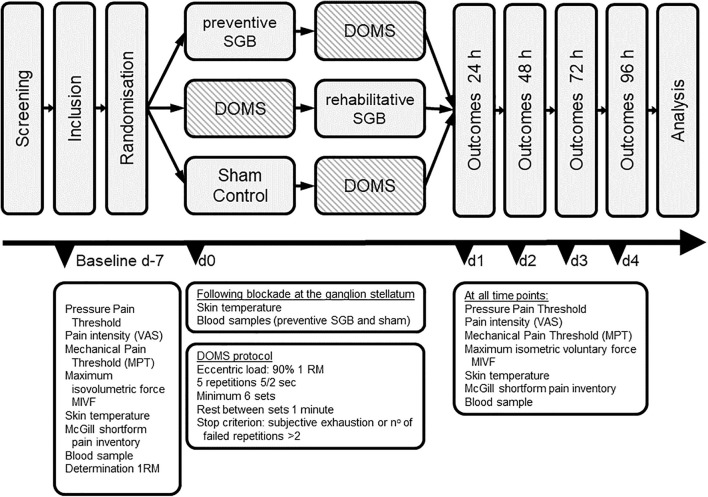
Trial design. Baseline measures were assessed at 7 days (d-7). After randomization, DOMS was induced at day 0 (d0), and outcomes were measured at indicated timepoints until 96 h after DOMS induction. SGB stellate ganglion block.

The recruitment was done through notices on the university campus and oral contact, and the participants did not have any conflicts of interest with the investigator or other parties. After signing written informed consent, subjects were randomly allocated by means of sequentially numbered opaque envelopes to one of three modalities: (i) Preventive SGB of the SNS, (ii) Rehabilitative SGB, and (iii) sham control. This random sequence was generated using the randomization program BiAS (version 1.00, epsilon-Verlag GbR Hocheim Darmstadt, Germany) and allocation was performed by the investigator in charge of the intervention. All participants and examiners were blinded to the intervention. DOMS was experimentally induced in all subjects, with volunteers receiving SGB (i) prior (preventive) or (ii) immediately after (rehabilitative) the induction of DOMS. Both, preventive and rehabilitative SGB were compared to sham control to investigate the effects. Baseline measures were assessed 1 week prior to the intervention. The total observation time was 96 h following the induction of DOMS (see Figure 1).

### Induction of Delayed-Onset Muscle Soreness

Delayed onset muscle soreness was experimentally induced at the non-dominant biceps muscle (and other flexors) using a standardized exercise protocol as previously described (Hubscher et al., 2008; Fleckenstein et al., 2016, 2017a,b). To determine their individual one repetition maximum (1 RM; i.e., the maximum weight lifted with one concentric contraction), all participants were seated at a preacher bench (Diagnos+© Schnell© Trainingsgeräte GmbH, Peutenhausen, Germany) and performed isolated biceps curls with a dumbbell at the screening visit. The 1 RM was determined for the elbow flexors by loading the dumbbell with free weights in 0.5 kg increments. Participants were encouraged verbally to elicit their maximal effort.

The 90% of the previously determined 1 RM was then used 1 week later to provoke DOMS through eccentric contractions. For this, the experimenter lifted the dumbbell until the subject’s elbow was fully flexed, and the subject lowered the weight eccentrically as slowly as possible until the elbow was extended. Subjects performed six sets of concentric/eccentric actions of five repetitions or failure of more than two repetitions. For each eccentric contraction repetition, subjects were instructed to slowly lower the dumbbell from a fully flexed to a fully extended position over 5 s, controlled by the beats of a metronome. The concentric movement was performed without weight and time between eccentric loads was 2 s. Time between sets was 1 min.

### Sample Size Estimation

The sample size estimation has been made on the basis of previous results (Fleckenstein et al., 2017b), showing a decrease in PPT at 48 h of approximately 25% (5.90 versus 4.39 kg/cm^2^). Taking into account that a clinically meaningful effect should not alter the PPT more than 10% and that a total of 5 measures will be performed, it will be necessary to treat 14 subjects to show such a difference when compared to control. On the rationale that both interventions will be compared to sham, each modality will contain 14 subjects. Due to high compliance observed in previous studies, a drop-out ratio of 9% is justifiable. Thus, the total sample size has been estimated *n* = 45.

### Intervention

The procedure of performing a block at the stellate ganglion has been previously described (Fischer et al., 2013; Puente de la Vega Costa et al., 2016), and was performed as a single shot blockade of the stellate ganglion of the non-dominant extremity using the modified lateral technique according to Leriche-Fontaine/Dosch. The patients lay in a supine position on a treatment couch. The cervical spine was slightly extended and rotated by 45° to the contralateral side. The used sterile injection needle (Sterican 0.2 mm × 40 mm, B. Braun Melsungen AG, Melsungen, Germany) was mounted to a syringe containing procaine (1%, 5 ml, Steigerwald Arzneimittel GmbH, Darmstadt, Germany). The point between distal and medial third of the belly of the sternocleidomastoideus muscle was palpated, and together with underlying structures, i.e., carotid artery, internal jugular vein, and vagus nerve, brushed in medial and ventral direction. The injection site was above the tuberculum caroticum at the transverse process of the sixth cervical vertebra. The needle passed by the tuberculum (direction approx. 45°medial + dorsal + caudal) whilst palpating. Following bidirectional aspiration, the injection of 3–4 ml procaine was made in about 10–20 mm depth. The successful block was clinically proven by the appearance of the Horner-Trias, i.e., ptosis, miosis, and enophthalmos. In addition, the perfusion of the upper body quarter was detected in order to observe an increase in skin temperature of the forearm (vasodilatation).

### Blinding Procedure

The preparation of participants in the sham control was the same as for preventive SGB, however, they did not receive any form of injection. Participants lay down prior to the induction of DOMS in a supine position (see above). The area of injection was masked sterile with opaque adhesive drapes, which did not let the participant visually follow the procedure. Disinfection was performed, then the participant was told to take a deep breath and, when expirating, fingers pushed gently against the skin. The participant was told that a warm sensation of skin on the upper arm may occur and that a Horner Trias may happen. Temperature of the forearm was equally assessed. The medical doctor in charge of interventions was told to maintain the same empathy, wording, and attention to all participants. All outcome measures were performed by other study staff not attending the interventions, to avoid performance bias.

### Outcome Measures

Main outcome measure was the assessment of the pressure pain threshold (PPT). A mechanical pressure algometer (pdt, Rome, Italy) with a range between 2 and 20 kg was used to assess the PPT. The algometer was applied to a set of five equidistant points, perpendicular to the belly of the biceps brachii muscle on a thought line between the tuberositas radii and the coracoid prominence, with increasing force at a rate of approximately 1 kg/cm^2^/s until the subject reported painful sensation, and the according force value was recorded (kg/cm^2^). Each point was measured three times, with 10 s intervals between trials, and the mean of all 15 (5 points × 3 times) values was used for the analysis. The reliability of this method has been shown previously (Nussbaum and Downes, 1998).

Secondary outcomes included the following:

#### Mechanical Pain Thresholds (MPT)

With a set of seven weighted pinprick stimulators each with a blunt contact area of 0.25-mm diameter, the MPTs are measured (MRC Systems GmbH, Heidelberg; Germany) (Chan et al., 1992). The intensity of the punctate stimulator’s increments increases by a factor of 2 from 8 to 512 mN. The method of limits is used to determine the intensity at which subjects distinguish between prick and blunt touch. The MPT tests for A-delta fiber mediated hyper- or hypo-algesia to pinprick stimuli. The MPT is applied to a set of five equidistant points (see above).

#### Pain Intensity

Pain intensity at rest and during active movement of the biceps muscle is assessed using a visual analog scale VAS ranging from 0 to 10 cm (with 0 indicating no pain and 10 experiencing the worst imaginable pain). Participants completed the Short Form McGill Pain Questionnaire (SF-MPQ) for sensations experienced in the non-dominant biceps muscle. The SF-MPQ is adapted to the German language comprising eleven sensory and four affective pain descriptors that could be ranked in intensity from 0 = none to 3 = severe (Melzack, 1987). The sum of ranked values provides a sensory (SPRI; 0–33), an affective (APRI; 0–12), and a total pain score (TPRI; 0–45).

To obtain maximum isometric voluntary force (MIVF), the m3 (multi muscle machine) Diagnos+© (Schnell© Trainingsgeräte GmbH, Peutenhausen, Germany) is used. All measures are performed in a respective standardized seated position, all participants are positioned on the seat to avoid auxiliary muscle contraction. Three tests per muscle are performed with contractions lasting 5 s, separated by 2 min rest intervals. Force × time is displayed on a screen providing an immediate feedback. In addition, participants are verbally encouraged in a standardized order to elicit maximal effort. Sufficient test–retest reliability and construct validity has been shown for this test (Herbsleb et al., 2010).

#### Biomarker

cfDNA has been proposed to be relevant in sports-related settings, showing associations with muscular load (Haller et al., 2018) and overload (Fatouros et al., 2006). Thus, we decided to investigate this biomarker as a potential predictor in the occurrence of DOMS and EIMD additionally. Blood samples were collected before and after local anesthesia, immediately after the induction of DOMS and at 10, 20, 30, and 60 min thereafter. Venous blood was taken at the cubital vein of the ipsilateral arm and centrifugated at 3,000 rpm for 10 min. Plasma samples were stored at −20°C. The cfDNA was measured at the Department of Sports Medicine, Johannes Gutenberg University Mainz as described by Neuberger et al. (2021). Briefly, after a 1:10 dilution of the plasma samples in UltraPure^TM^ DNase/RNase-Free H2O (Invitrogen, Corporation, CA, United States), a pre-validated qPCR assay was used to amplify a hominoid specific repetitive element, which allows sensitive and reliable quantification of cfDNA in low amounts of plasma samples.

To assure stellate block accuracy, the Horner trias and skin temperature among participants were assessed. The Horner trias includes miosis, ptosis, and enophthalmos of the eye and was rated as yes = apparent, no = not apparent, or unclear. The skin temperature at the forearm as well as the upper arm of the respective side was monitored with a commercially available infrared thermometer (Voltcraft^®^ IR 260-8S, Wollerau, Switzerland). Temperature was monitored before and after the intervention.

### Statistics

Baseline characteristics were analyzed with ANOVA (for continuous measures) and chi-square test (for nominal data) to assess for differences among the three study modalities.

Statistical analysis was conducted for comparison of the primary and secondary outcome measures between each of either preventive or rehabilitative SGB and sham control. No evidence was found that the parametric tests used were inappropriate. As data is longitudinal, we applied a mixed-effects analysis, i.e., a 4 × 2 model (time × group [SGB and sham]) to analyze the effects on pressure pain threshold, force, and pain intensity with the levels at 24, 48, 72, and 96 h after the induction of DOMS compared to baseline.

Data was analyzed according to Mauchly’s test for sphericity and the Huynh–Feldt correction was used in case sphericity was not present. If statistically significant, the mixed-model used for each outcome variable was followed by *post hoc* pairwise comparisons of change scores between each of the four time points and baseline. The level of significance was achieved at *p* < 0.05. Effect sizes according to Cohen were calculated for all relevant outcomes.

Demographic data are presented as mean ± standard deviation (SD), whereas all other data and all parametric data are displayed as mean ± standard error of the mean (SEM).

Data analysis was performed with the SPSS statistical software system, version 21.0 (SPSS Inc., Chicago, IL, United States).

## Results

We included 45 participants (female 11, male 34, age 24.16 ± 6.67 years [range 18–53], height 178.13 ± 8.95 cm; weight 73.94 ± 10.54 kg; BMI 23.22 ± 2.09 kg/m^2^). No drop-out occurred during the study. Participants were randomly allocated to three modalities of *n* = 15 each. Muscle soreness was familiar to all participants, with the majority perceiving DOMS weekly (*n* = 12) or monthly (*n* = 29). The mean training load was 5.13 ± 2.53 h/week and 1.42 ± 1.68 h/week of endurance training. Forty-two participants performed some sports regularly. The 1RM did not differ between groups (Kruskal–Wallis *p* = 0.629). All participants completed the full six sets of DOMS induction. There were no significant demographic differences (Table 1).

**TABLE 1:** Demographic data, except 1RM data are presented as mean (SD), 1RM as median (95%-CI).

	Rehabilitative SGB	Preventive SGB	Sham control	Total	*p*-value
*Sex (m/f)*	2/13	6/9	3/12	11/34	0.209
*Age (years)*	22.2 (2.93)	27.6 (10.2)	22.67 (2.79)	24.16 (6.67)	0.181
*Height (cm)*	180.27 (7.35)	177 (8.94)	177.13 (10.5)	178.13 (8.95)	0.538
*Weight (kg)*	75.77 (8.19)	72.27 (13.56)	73.8 (9.56)	73.94 (10.54)	0.670
*BMI (kg/m^2^)*	23.29 (1.85)	22.88 (2.58)	23.48 (1.85)	23.22 (2.09)	0.731
*Main sports^1^/training, load*					0.125
• *Fitness*		5	5	10	
• *Soccer*	3	5	3	11	
• *Hockey*	11	3	6	20	
• *Cycling*		1		1	
*Training load (h/w)*	5.53 (2.68)	4.57 (2.32)	5.3 (2.64)	5.13 (2.53)	0.247
*Endurance training (h/w)*	1.87 (1.68)	1.03 (1.61)	1.37 (1.75)	1.42 (1.68)	0.220
*muscle soreness^2^*					0.283
• *Never*					
• *1–2/year*	1	13	2	3	
• *Monthly*	9	2	7	29	
• *Weekly*	5		5	12	
• *>2/week*			1	1	
*1RM (kg)*	23.0 (19.1–26.8)	23.5 (12.7–25.0)	25.0 (18.2–28.2)	24.3 (18.9–24.4)	0.629

*^1^Training load indicates the weekly time that is spent performing a main sport activity.*

*^2^Muscle soreness was assessed on a fivefold Likert scale ranging from 0 = never to 4 = more than 2 times a week regardless of the muscle group affected.*

In the assessment of the stellate block accuracy, a Horner trias (yes/no/unclear) was found in 13/1/1 of preventive SGB, in 12/3/0 of rehabilitative SGB, and 1/13/1 of the sham control (*p* < 0.001). The participants’ skin temperature was increased in all but one (93%) of preventive SGB, 90% of rehabilitative SGB (with *n* = 2 unchanged at the upper arm), and 80% of the sham control (forearm *p* = 0.508/upper arm *p* = 0.146). The mean increase in skin temperature at the upper arm was 0.92 ± 1.05°C (preventive), 0.73 ± 0.42°C (rehabilitative), 0.45 ± 0.54°C (sham), and at the forearm 1.12 ± 1.19°C (preventive), 0.60 ± 0.89°C (rehabilitative), 0.45 ± 0.61°C (sham), all without statistical significance.

The DOMS was successfully induced as eccentric exercise led to a decrease in the sham control of the pressure pain threshold at 24 h (Δ−0.74 ± 0.51 kg/cm^2^; *p* < 0.001), 48 h (Δ−0.71 ± 0.69 kg/cm^2^; *p* = 0.001) and 72 h (Δ −0.44 ± 0.71 kg/cm^2^; *p* = 0.030), as well as an increase of the pain intensity at rest at 24 h (Δ 1.02 ± 1.41 cm VAS; *p* = 0.014) and 48 h (Δ 1.02 ± 1.41 cm VAS; *p* = 0.014), and the pain intensity when moving the arm at 24 h (Δ 2.70 ± 2.36 cm VAS; *p* = 0.001), 48 h (Δ 2.05 ± 1.51 cm VAS; *p* < 0.001), 72 h (Δ 1.01 ± 1.18 cm VAS; *p* = 0.005), and 96 h (Δ 0.34 ± 0.41 cm VAS; *p* = 0.007). See Table 2 for mean ± SEM values. The decline in MIVF at 24 h was close to significance (Δ 3.40 ± 6.20 cm VAS; *p* = 0.052), altogether indicating that DOMS was induced but muscle damage (EIMD) was only marginally generated.

**TABLE 2:** Outcome measures.

		Rehabilitative SGB	Preventive SGB	Sham control	Total
*PPT (kg/cm^2^)*	Baseline	4.68 ± 0.98	4.12 ± 0.82	4.95 ± 1.6	4.58 ± 1.21
	24 h	3.94 ± 0.96	3.47 ± 0.84	4.21 ± 1.53	3.87 ± 1.17
	48 h	4.08 ± 1	3.75 ± 1.08	4.24 ± 1.39	4.02 ± 1.16
	72 h	4.3 ± 1.12	3.75 ± 0.89	4.51 ± 1.62	4.19 ± 1.26
	96 h	4.67 ± 1.03	4.17 ± 0.86	5.3 ± 1.67	4.71 ± 1.29
*VAS at rest*	Baseline	0.04 ± 0.08	0.01 ± 0.03	0 ± 0	0.02 ± 0.05
	24 h	0.81 ± 0.68	0.65 ± 0.68	1.02 ± 1.41	0.83 ± 0.97
	48 h	0.81 ± 0.69	0.51 ± 0.52	0.43 ± 0.56	0.58 ± 0.6
	72 h	0.49 ± 0.53	0.32 ± 0.34	0.2 ± 0.39	0.34 ± 0.43
	96 h	0.19 ± 0.24	0.17 ± 0.39	0.05 ± 0.11	0.14 ± 0.27
*VAS at motion*	Baseline	0.11 ± 0.21	0.02 ± 0.06	0.01 ± 0.05	0.05 ± 0.14
	24 h	2.05 ± 1.35	1.55 ± 0.9	2.71 ± 2.36	2.11 ± 1.68
	48 h	2.33 ± 1.58	1.11 ± 0.83	2.06 ± 1.52	1.83 ± 1.43
	72 h	1.23 ± 0.9	0.8 ± 0.73	1.03 ± 1.18	1.02 ± 0.95
	96 h	0.49 ± 0.53	0.37 ± 0.43	0.35 ± 0.41	0.4 ± 0.45
*MIVF (N)*	Baseline	62.33 ± 19.7	49.87 ± 23.94	62.87 ± 19.95	58.36 ± 21.66
	24 h	61.13 ± 19.96	47.67 ± 22.24	59.47 ± 22.41	56.09 ± 21.92
	48 h	62.93 ± 20.58	49 ± 22.21	59.87 ± 21.92	57.27 ± 21.94
	72 h	64.33 ± 20.89	51.47 ± 22.28	62.53 ± 20.84	59.44 ± 21.63
	96 h	66.87 ± 21.01	53 ± 22.47	63.67 ± 21.39	61.18 ± 21.97
*MPT (mN)*	Baseline	174.86 ± 76.44	144.19 ± 82.21	124.92 ± 75.27	147.99 ± 79.02
	24 h	154.63 ± 74.86	128.13 ± 94.87	134.77 ± 104.68	139.18 ± 90.9
	48 h	157.57 ± 101.53	140.06 ± 109.26	128.06 ± 106.46	141.89 ± 104.09
	72 h	136.88 ± 89.62	149.32 ± 110.5	148.2 ± 121.4	144.8 ± 105.65
	96 h	163.56 ± 116.51	161 ± 116.93	168.41 ± 115	164.32 ± 113.52
*TPRI (0–45)*	Baseline	0.2 ± 0.41	0 ± 0	0 ± 0	0.07 ± 0.25
	24 h	4 ± 3.59	6.6 ± 5.62	7.2 ± 5.19	5.93 ± 4.97
	48 h	4.47 ± 3.91	4.53 ± 2.92	5.6 ± 3.68	4.87 ± 3.49
	72 h	3.07 ± 2.89	2.47 ± 2.2	3.67 ± 3.27	3.07 ± 2.8
	96 h	1.13 ± 1.19	1.4 ± 1.96	1.2 ± 1.42	1.24 ± 1.52

**FIGURE 2:**
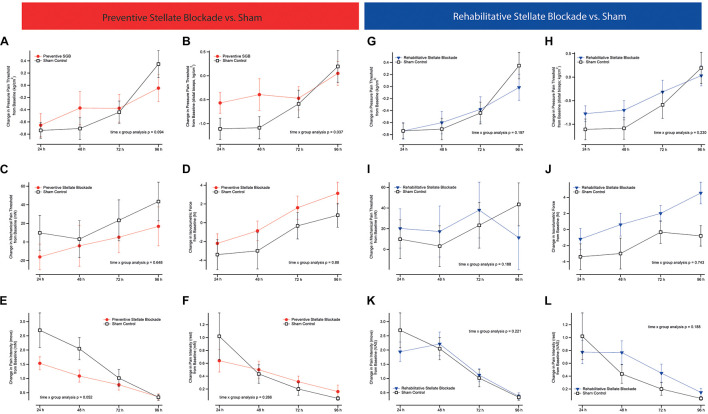
Pain outcomes. Figures comparing changes in functional and pain related outcomes between either preventive or rehabilitative SGB, and sham control. **(A,G)** Overall pressure pain threshold (PPT) as the mean of five measures equidistant along the upper biceps muscle, **(B,H)** PPT above the distant belly of the biceps muscle, **(C,I)** mechanical pain threshold, **(D,J)** maximum isovolumetric force of the biceps muscle, **(E,K)** pain intensity of the biceps muscle whilst bending the arm, **(F,L)** pain intensity of the upper arm at rest. All analyses are time x group analysis indicating between group changes over time. Data are presented as mean ± SEM.

Time × group (4 × 2 model [SGB and sham]) analysis of the main outcome did not reveal a significant difference in the alteration of pressure pain threshold when comparing preventive SGB to sham (*p* = 0.094, Figure 2A) or rehabilitative SGB to sham (*p* = 0.197, Figure 2G). Focusing on the muscular area with the greatest eccentric load (distal fifth, see Figure 2B), there was a significant time x group interaction comparing preventive to sham (*p* = 0.034, Figure 2B), but no comparing rehabilitative to sham (*p* = 0.230, Figure 2H).

Time × group (4 × 2) analysis did not reveal significant differences in the alteration of mechanical pain threshold (*p* = 0.665, Figure 2C), pain intensity at rest (*p* = 267, Figure 2F), quality of pain (TPRI *p* = 0.574, SPRI *p* = 0.555, APRI *p* = 0.554), or isovolumetric force (*p* = 0.880, Figure 2D) when comparing preventive SGB to sham. There was a significant effect on pain at motion (*p* = 0.048, Figure 2E), with *post hoc* statistical difference at 48 h (Δ1.09 ± 0.82 cm VAS vs. Δ2.05 ± 1.51 cm VAS; *p* = 0.04).

There were no significant differences in the alteration of mechanical pain threshold (*p* = 0.188, Figure 2I), pain intensity at rest (*p* = 0.239, Figure 2L) and in motion (*p* = 0.221, Figure 2K), quality of pain (TPRI *p* = 0.070, SPRI *p* = 0.147, APRI *p* = 0.068), or isovolumetric force (*p* = 0.743, Figure 2J) when comparing rehabilitative SGB to sham.

**FIGURE 3:**
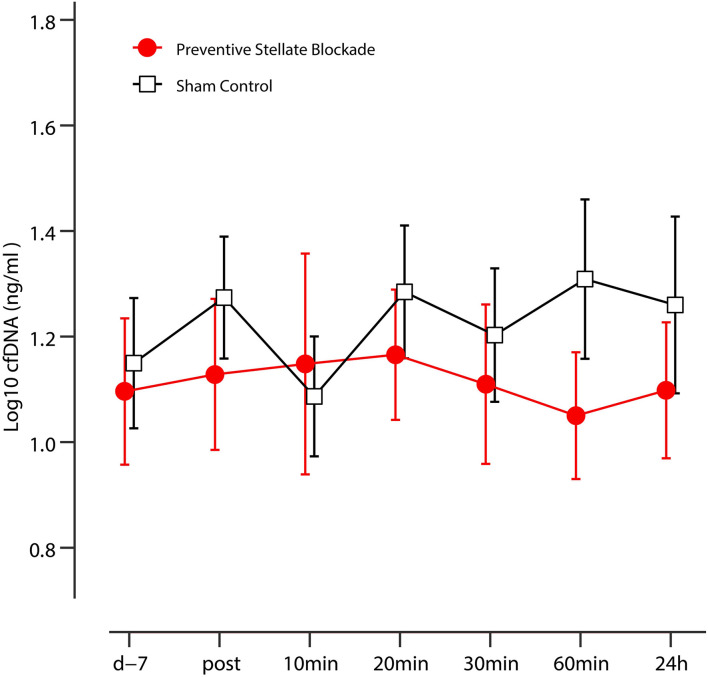
Kinetics of the plasma cfDNA in sham control and preventive SGB. Data are presented as mean, error bars indicate 95% CIs.

On an explorative basis, we evaluated the role of cfDNA as a potential molecular marker of DOMS in preventive SGB and sham control. DOMS led to an increase in venous cfDNA within the first 24 h after eccentric exercise (time effect *p* = 0.018), with a peak at 20 and 60 min (see Figure 3 and Table 3). After 60 min, cfDNA scores following preventive SGB were significantly decreased compared to sham (unpaired *t*-test *p* = 0.008). At both times, 20 and 60 min, cfDNA significantly correlated with observed changes in PPT (Table 4). In the sham control, the increase in cfDNA predicted decreased thresholds at 72 h (*r* = −0.55; *p* = 0.03, *r* = −0.51, *p* = 0.05). The 20-min increase was more sensitive, as it tended toward significance at 48 h (*r* = 0.44; *p* = 0.1) and predicted the early decrease of PPT following preventive SGB at 24 h (*r* = 0.53; *p* = 0.04).

**TABLE 3:** cfDNA levels within the first 24 h following the induction of DOMS.

	preventive SGB		Sham control	
	Mean ± SD	Change %	Mean ± SD	Change %
*Baseline*	12.47 ± 1.74	0.00	14.11 ± 1.67	0.00
*0 min*	13.44 ± 1.72	7.75	18.79 ± 1.62	33.11
*10 min*	14.06 ± 2.3	12.75	12.21 ± 1.6	–13.50
*20 min*	14.64 ± 1.67	17.41	19.26 ± 1.69	36.46
*30 min*	12.88 ± 1.83	3.23	15.95 ± 1.66	13.02
*60 min^#^*	11.23 ± 1.65	–10.00	20.36 ± 1.87	44.29
*24 h*	12.54 ± 1.71	0.53	18.19 ± 2.01	28.90

*^#^Preventive vs. sham, *p* = 0.008.*

**TABLE 4:** Correlations between cfDNA levels and pain outcomes.

		Preventive SGB @20 min	Sham control @20 min	Preventive SGB @60 min	Sham control @60 min
Δ*PPT*	24 h	***r* = −0.53; *p* = 0.04**	*r* = 0.06; *p* = 0.84	*r* = −0.22; *p* = 0.43	*r* = −0.13; *p* = 0.65
	48 h	*r* = −0.17; *p* = 0.54	***r* = −0.44; *p* = 0.1**	*r* = −0.2; *p* = 0.47	*r* = −0.18; *p* = 0.52
	72 h	*r* = −0.41; *p* = 0.13	***r* =** −**0.55; *p* = 0.03**	*r* = −0.37; *p* = 0.18	***r* =** −**0.51; *p* = 0.05**
	96 h	*r* = −0.22; *p* = 0.42	*r* = −0.2; *p* = 0.46	*r* = −0.06; *p* = 0.82	*r* = 0.01; *p* = 0.98
Δ*VASmotion*	24 h	*r* = 0.1; *p* = 0.72	*r* = 0.24; *p* = 0.4	*r* = 0.3; *p* = 0.28	*r* = −0.1; *p* = 0.73
	48 h	*r* = −0.09; *p* = 0.75	*r* = 0.24; *p* = 0.4	*r* = −0.11; *p* = 0.7	*r* = −0.17; *p* = 0.55
	72 h	*r* = 0.04; *p* = 0.89	*r* = 0.32; *p* = 0.25	*r* = 0.11; *p* = 0.69	*r* = −0.01; *p* = 0.98
	96 h	*r* = 0.06; *p* = 0.82	*r* = 0.43; *p* = 0.11	*r* = 0.14; *p* = 0.62	*r* = −0.07; *p* = 0.82
Δ*VASrest*	24 h	*r* = 0.41; *p* = 0.13	*r* = −0.05; *p* = 0.87	***r* = 0.61; *p* = 0.02**	*r* = −0.43; *p* = 0.11
	48 h	*r* = 0.16; *p* = 0.56	*r* = 0.32; *p* = 0.24	*r* = 0.31; *p* = 0.26	*r* = −0.15; *p* = 0.59
	72 h	*r* = 0.09; *p* = 0.75	*r* = 0.18; *p* = 0.53	*r* = 0.25; *p* = 0.38	*r* = 0.02; *p* = 0.95
	96 h	*r* = 0.32; *p* = 0.24	*r* = 0.1; *p* = 0.74	***r* = 0.48; *p* = 0.07**	*r* = 0.02; *p* = 0.93
Δ*MIVF*	24 h	*r* = −0.12; *p* = 0.66	*r* = 0.16; *p* = 0.57	*r* = 0.07; *p* = 0.81	*r* = 0.31; *p* = 0.25
	48 h	*r* = 0.05; *p* = 0.86	*r* = −0.04; *p* = 0.88	*r* = 0.15; *p* = 0.6	*r* = 0.1; *p* = 0.71
	72 h	*r* = −0.08; *p* = 0.79	*r* = 0.28; *p* = 0.32	*r* = 0.28; *p* = 0.32	*r* = 0.34; *p* = 0.22
	96 h	*r* = 0.07; *p* = 0.79	*r* = 0.14; *p* = 0.62	*r* = 0.22; *p* = 0.43	*r* = 0.25; *p* = 0.36

*Significant correlations are shown in bold.*

We could not detect any gender differences.

## Discussion

We present a translational study that aimed to investigate if sympathetic activity impacts the occurrence and intensity of sports-related acute pain. From a statistical point of view, we could not detect statistically significant effects of preventive or rehabilitative SGB following the induction of DOMS by means of eccentric exercise of the upper limb. From a clinical point of view, one might assume that the preventive block did reduce the impact of DOMS to some extent, as shown by decreased pain intensity, a trend toward significance in PPT (*p* < 0.01), and reduced plasma markers, i.e., cfDNA. According to the assessment of pain intensity, the largest effects could be observed at 48 h, and effect sizes at this time suggest moderate impact, i.e., pain at motion *d* = 0.79, pain at motion d 0.51 PPT *d* = −1.5. Consequently, we cannot rule out that decreasing the sympathetic activity at an early stage (preventive) might be appropriate to enhance recovery following DOMS.

Furthermore, this study gives hints for molecular changes induced by interrupting the sympathetic reflex. The increase of plasma cfDNA was remarkably reduced in the preventive stellate blockade group compared to sham. The calculated effect size at 60 min, *d* = 5.18 corresponds to a huge effect (Sawilowsky, 2009). As cfDNA seems to be a correlate of the decrease of PPT as a sign of increased pain perception, it appears that the cfDNA describes the alleged shift in recovery from DOMS toward an earlier time point following preventive stellate blocks. Still, these results remain exploratory and hypothetical, but should be subject to further research. If reproducible, cfDNA could present for the first time a reliable marker of acute pain.

In addition, our results reveal some of the complex role of sympathetic control in DOMS and EIMD. In this context, it is important to remember that the mode of control over sympathetic and parasympathetic innervations may be reciprocal, non-reciprocal, or uncoupled (Berntson et al., 1991). Thus, changes in sympathetic excitability may also affect parasympathetic tone. In this study we performed SGB to interrupt the central and peripheral reflex pathways of the SNS. In this context it is the physiologic understanding of the SGB, as briefly explained in the following section, that allows profound insights into the molecular changes induced by interrupting the sympathetic reflex. The short-term outcome during the duration of action of the local anesthetic then tells how much the part of the “sympathetically maintained pain” and inflammation is. After the temporary disruption, the systems have a chance to reorganize (Fischer, 2017). We have postulated this as the main mechanism of action in the long-term effect on pain and inflammation (Egli et al., 2015). As reviewed by Fischer et al. (2021), pro-inflammatory cytokines decrease, and anti-inflammatory cytokines increase after an SGB. In addition, SGB can regulate endothelial dysfunction, microcirculation, and neurogenic edema. The latter also appear to play an important role in DOMS (Wilke et al., 2017). Moreover, the SBG affects the immune system by modifying the distribution of lymphocyte subsets and natural-killer cell activity (Yokoyama et al., 2000). Consequently, our model is solid enough to reproduce some of the suggested effects in experimentally-induced DOMS *in vivo*.

**FIGURE 4:**
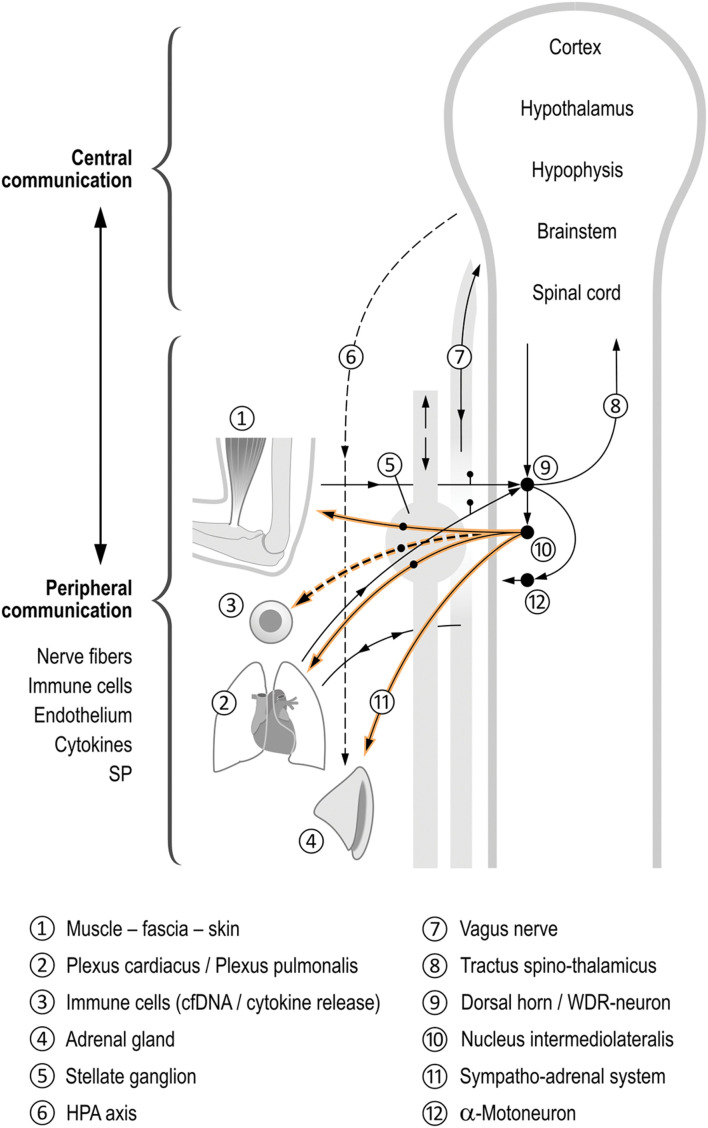
Coordinating function of the autonomic nervous system (ANS) in the inflammatory process. This figure schematically shows the different control by sympathetic regulation of physiological processes following, for example, muscular overload of the upper extremity ➀. Spinal reflexes cause an activation of the stellate ganglion activity➄, but also activate different neuronal and non-neuronal feedback loops at different levels, namely the peripheral–spinal, brainstem, and brain, all being interdependent (Jänig, 2006; Janig, 2014; Fischer et al., 2021), see Figure balls 6–12. They coordinate the musculoskeletal system, internal organs, and immune and inflammatory systems (balls 1–4). The nervous system–immune system communication is obvious and occurs in both the peripheral and central parts of the ANS (Elenkov et al., 2000; Bellinger and Lorton, 2014; Janig, 2014). We hypothesize a release of cfDNA to be linked to an increased sympathetic activity. During muscle work, all systems shown here can be activated, presumably depending on the number of muscles actuated. The influence of the SNS on inflammation and pain is interdependent (for details see manuscript). Afferents lead as sensory nerve fibers from the musculoskeletal system, the skin, and from the internal organs to the spinal cord (Sandkühler, 2013). There, they converge at the same multireceptive posterior horn neurons (wide dynamic range neurons WDR) (Janig, 2014). The transmission of these impulses now occurs divergently via the spinothalamic tract into the brain where reflex arcs are “closed” (via hypothalamus and RVLM). Another reflex arc is completed in the spinal cord itself: from the posterior to the anterior horn (motoneurons) via interneurons on the one hand, as well as to the sympathetic intermediolateral nucleus in the lateral horn. From this nucleus, divergent sympathetic efferents travel simultaneously to the skin, the musculoskeletal system, and the metamerically associated internal organ (Fischer, 2017). The sympathetic centers in the brain (e.g., hypothalamus and RVLM) also receive information indirectly from sensory fibers of the vagus nerve (Tracey, 2002). The vagus nerve projects, among others, from the heart and lungs into areas in the brainstem (nucleus of the solitary tract SN). This in turn establishes connections to the sympathetic core areas in the brain and the hypothalamus (Ross et al., 1985; Affleck et al., 2012), closing yet another feedback loop. Thus, sympathetic efferent impulses are also dependent on vagal input (e.g., from the heart and lungs). However, they are also dependent on, for example, cytokine concentration in the periphery, which is reported to the brain (Schaible et al., 2011; Chiu et al., 2013; Ji et al., 2016).

The effects on inflammation especially, as shown by the alteration of cfDNA kinetics, seem relevant in regard to the role of the ANS (especially the SNS) in relation to pain, inflammation, and processes in the immune system as well as microcirculation. On the one hand, several mediators have been shown to promote DOMS, including cytokines such as IL-6 (Robson-Ansley et al., 2010), TNF-α (Dos Santos et al., 2020), immune cells (Zelenka et al., 2005), and proinflammatory neuropeptide substance P (SP) (O’Connor et al., 2004) and other pathways, including the B2 bradykinin receptor–nerve growth factor (NGF) pathway and activation of the COX-2-glial cell line derived neurotrophic factor (GDNF) pathway (Mizumura and Taguchi, 2016). On the other hand, these substances have been shown to exert neuromodulation (Ji et al., 2016), allowing communication between the ANS and the immune system. The systems are complex due to their interdependency: tissue damage releases SP from sensory nerve fibres, which in turn stimulate cytokine production in immune cells (Ansel et al., 1993; Hosoi et al., 1993; Stanisz, 2001). This mediates a simultaneous alteration of the microcirculation, and plasma extravasation and edema occur. Neurogenic inflammation has developed, in which the ANS, especially the SNS, plays an important role (for review see Figure 4). As a result, both pain and inflammation are amplified with additional activation of the sympathetic nervous system (e.g., stress, circulatory regulation, etc.). The reflex action can be further sensitized via so-called synaptic long-term potentiation (LTP) at the level of the spinal cord in the WDR neurons (Sandkühler, 2000) and also in the sympathetic ganglia (Alkadhi et al., 2005). This LTP results in potentiation of the postsynaptic response to recurring constant stimulation. Thus, the sympathetic nervous system can store stimuli engrammatically, and responds to recurring physiological stimuli with an overshooting pathological response. In addition, the sympathetic nervous system contributes to the peripheral sensitization of nociceptors. In simple terms, we are dealing -as shown- with “sympathetically maintained pain and inflammation,” i.e., a communication between the nervous and immune systems, in which efferent (mainly sympathetic) and sensory nerves as well as messenger substances such as cytokines or cfDNA are involved. Feedback occurs peripherally, spinally, and in the brain.

Returning to our results, these circuits mean that SGB is able to interrupt the circuits at the upper extremity (biceps muscle) that are necessary for the induction of DOMS.

Hematopoietic cells are the major source of cfDNA in the circulation at rest and during exercise (Lui et al., 2003; Tug et al., 2015). Methylation specific analysis indicated that ∼55% of the total amount of plasma DNA originate from immune cells (Moss et al., 2018). Especially, neutrophils, which are the first line of immune defense, have been considered to be associated with an active release of cfDNA (Beiter et al., 2015). During inflammation the process of NETosis leads to the release of nuclear DNA and the production of cytokines. Whilst the defense of pathogens is the most described effect of NETosis, an expanding body of research associates NETosis with non-infectious organ injury (Cahilog et al., 2020). Acute inflammation, as a result of DOMS-induced exercise, leads to a recruitment of neutrophils into injured muscles (Macintyre et al., 2000) which might be associated with an active release of cfDNA.

The increase of plasma cfDNA was remarkably reduced comparing preventive SGB to sham. The calculated effect size at 60 min, *d* = 5.18 corresponds to a huge effect (Sawilowsky, 2009). As cfDNA seems to be a correlate of the decrease of PPT as a sign of increased pain perception, it appears that the cfDNA describes the alleged shift in recovery from DOMS toward an earlier time point following preventive stellate blocks which might be associated with the recruitment of immune cells and their activation. Although the physiological association between pain and cfDNA remains elusive, associations between pain and cfDNA are in line with observations in sickle cell disease (Al-Humood et al., 2014) and lumbar degenerative disease (Hiyama et al., 2021). In this context it is signaling multiprotein complexes, called inflammasomes, being part of the innate immune system and that are fundamentally linked to the neuro-inflammatory mechanisms that are contributing to painful pathologies (Starobova et al., 2020). Still, these results remain exploratory and hypothetical, but should be subject to further research. If reproducible, cfDNA could present for the first time a reliable marker of acute pain.

Although the SNS usually has a proinflammatory effect, it also has -rarely and in later phases- an anti-inflammatory effect (Pongratz and Straub, 2014; Strong et al., 2020). Therefore, the timing of an SGB is important (Schaible and Straub, 2014). In our experience, the earlier it is performed, the greater its anti-inflammatory effect.

Our study design only involved the exhaustion of a singular muscle group. As shown in Figure 4, the degree of “sympathetic response” to a stimulus depends not only on the peripheral-spinal reflex component but also on how many stimulus impulses generally reach the autonomic centers in the brain. We hypothesize that the sympathetic impact on the DOMS response to a muscular effort involving a single muscle (in this study biceps) is smaller compared to the involvement of a larger number of muscles (running, tennis, etc.). In the latter, more stimuli will reach the autonomic centers: on the one hand via the sympathetic system (sensory fibers via the truncus sympathicus), on the other hand via sensory fibers in the vagus nerve (whose projection zones in the brain include connections with sympathetic centers, see above). Consequently, there is an increased sympathetic efferent “output” via hypothalamus and RVLM. This largely systemic sympathetic output includes the SA system and sympathetic efferents and can in turn accelerate the inflammatory cascade via increased production of cytokines and SP (see above). This mediates changes in the microcirculation and interstitium (including ph-value) and thereby increased sensitization of nociceptors. In addition, it seems plausible that the above-mentioned coupling phenomena and LTP have a greater effect because of the activated sympathetic efferents. The increased sympathetic “outflow” would be additionally amplified by kinds of sports that involve psychological stress (also influencing hypothalamus and RVLM) besides the whole-body load, such as a tennis competition or climbing. Still, it is hard to differentiate the proinflammatory effect of this sympathetic output through its partial inhibition by the systemic activation of the HPA axis.

Both acute and chronic exercise increase pain. For example, exhaustive endurance exercise is followed by a decrease in tissue pressure pain threshold, thus a hyperalgetic condition (Krüger et al., 2016). Repetitive stress, as induced by forced swimming, has been shown to induce thermal hyperalgesia by diminishing central serotonin activity (Quintero et al., 2000). Still, hyperalgesia seems to occur in exhaustive states of exercise (i.e., maximum of stress) (Kortenjann et al., 2020), and moderate physical activity is an important component of effective chronic pain management (Rice et al., 2019). When looking at this interaction it is also necessary to pay attention to the HPA-axis which has a potential anti-inflammatory effect via cortisol (thus reducing cytokines). In this regard, it is important to distinguish adaptive short-term stress from maladaptive responses to pain, as it may intensify cortisol secretion and condition a sensitized physiologic stress response perpetuating cortisol dysfunction (Hannibal and Bishop, 2014). However, this effect does not seem to cancel out the inflammatory effect, especially in the untrained. Still, there are hints that the stress response between trained and untrained persons may differ (Calle and Fernandez, 2010; Olesen et al., 2015). As 41 of 45 participants in our study were familiar to DOMS and were sports active, our results therefore suggest that expected autonomic regulation in untrained individuals may be even greater.

Finally, when compared to previous studies (Fleckenstein et al., 2016, 2017a,b), our DOMS protocol resulted in maximum pain intensities of about 50% of normally achieved scores. In addition, we could not detect significant changes in muscular force indicating less substantial exercise-induced damage. As many of our participants were parts of competitive sports squads, we cannot rule out that myoprotective adaptations in terms of repetitive bout effects impeded enhanced muscle damage (Hyldahl et al., 2017). We determined the 1-RM maximum 1 week prior to the induction of DOMS, which may also have facilitated mechanism of adaptation. Twenty-nine of our participants reported to perform sports at least monthly and the mean combined training load was 6.5 h/week. Furthermore, our protocol comprised six sets with five repetitions each. Other labs report a larger number of sets and repetitions (Lau et al., 2015) or, as performed previously, exercise until exhaustion (Fleckenstein et al., 2017a).

### Limitations and Technical Considerations

When assessing SMP, the quality of the SGB must be ensured. It relies mainly on the Horner symptom complex and the temperature increase of the equilateral upper extremity during the duration of action of the local anesthetic. This temperature increase is important because the sole Horner’s symptom complex can also occur following an injection to the cervical sympathetic trunk cranially to the stellate ganglion. We made sure that both conditions were met in our study. Overall, the block seemed to be sufficient, although we cannot rule out that intervention failed in four participants, and we cannot explain the one sham participant appearing with a Horner trias. The mean skin temperature increased in >80% of participants, with descriptively higher increases following intervention. The infrared thermography has a systematic error of 2%, thus we cannot assume the correctness of thermal measures. Removing doubtful participants from our dataset did not change the results of the analysis, thus we decided to fully report all datasets.

Participants in the control group experienced a touch of skin. We cannot exclude that this stimulus already exhibited relevant neurophysiologic (e.g., Olausson et al., 2002) and autonomic responses (for review: Sato et al., 1997; see Figure 4). Besides observation bias, this could partly explain the observed potential Horner sign in one of the control participants.

We are aware that the role of cytokines in DOMS is not yet clear. We suppose cfDNA, besides being an inflammatory marker, to be a valuable diagnostic tool due to it fast kinetics and correlation with clinical outcomes. Still, other inflammatory biomarkers (Vadasz et al., 2020) as well as neurotrophic factors (Mizumura and Taguchi, 2016) have been discussed in this regard, which we did not assess.

As mentioned above, muscle soreness was achieved only in the isolated biceps muscle, and the SMP component was low. A whole-body load involving almost all muscles with marked cardiopulmonary involvement as well as simultaneous psychological stress would have brought a better result of the SGB with a higher SMP component. Whole-body exhaustion seems to be a more reliable approach when aiming to study the effects of exercise on neuromuscular fatigue (Wilke et al., 2016). This difference also applies, for example, to blood flow in muscles during exercise (Joyner and Casey, 2015).

## Conclusion

This study gives starting points for future studies to influence the sympathetic control of pain and inflammation following sports-related stress and supposed trauma. On a statistical basis, the effects of SGB on DOMS seem small, while from a clinical and experimental point of view several parameters indicate their role in the autonomic regulation. Further research is encouraged to replicate and more closely determine the observed effects.

## Data Availability Statement

The raw data supporting the conclusions of this article will be made available by the authors, without undue reservation, to any qualified researcher.

## Ethics Statement

The studies involving human participants were reviewed and approved by Ethics Committee of the University of Frankfurt. The patients/participants provided their written informed consent to participate in this study.

## Author Contributions

JF, PS, and WB contributed to conception and design of the study. JF, EN, AS, FC, and PB carried out the experiments. JF and PS performed the statistical analysis. JF wrote the first draft of the manuscript. JF, EN, PS, and LF wrote sections of the manuscript and prepared figures and tables. All authors contributed to manuscript revision, read, and approved the submitted version.

## Conflict of Interest

The authors declare that the research was conducted in the absence of any commercial or financial relationships that could be construed as a potential conflict of interest.

## Publisher’s Note

All claims expressed in this article are solely those of the authors and do not necessarily represent those of their affiliated organizations, or those of the publisher, the editors and the reviewers. Any product that may be evaluated in this article, or claim that may be made by its manufacturer, is not guaranteed or endorsed by the publisher.
